# Controlled expansion stent grafts versus legacy stent grafts for transjugular intrahepatic portosystemic shunt: a single-centre retrospective study on the incidence of hepatic encephalopathy

**DOI:** 10.1186/s42155-025-00557-8

**Published:** 2025-05-24

**Authors:** Afonso Fonseca, Rui Ramos, Élia Coimbra, António Caetano, Teresa Neves, Rafaela Pereira, Inês Conde Vasco, Marta Alves, Tiago Bilhim

**Affiliations:** 1 Interventional Radiology Unit, Curry Cabral Hospital, Unidade Local de Saúde São José, Centro Clínico Académico de Lisboa, Lisbon, Portugal; 2 Interventional Radiology Unit, Unidade Local de Saúde Trás-Os-Montes E Alto Douro, Vila Real, Vila Real, Portugal; 3 Epidemiology and Statistics Unit, Research Center, Unidade Local de Saúde São José, Centro Clínico Académico de Lisboa, Lisbon, Portugal; 4 Nova Medical School|Faculdade de Ciências Médicas da UNL, and Centro de Estatística E Aplicações da Universidade de Lisboa (CEAUL), Lisbon, Portugal

**Keywords:** Cirrhosis, Hepatic encephalopathy, Transjugular Intrahepatic Portosystemic Shunt, Variceal bleeding, Venous intervention

## Abstract

**Purpose:**

Assess incidence of hepatic encephalopathy (HE) after transjugular intrahepatic portosystemic shunt (TIPS) in patients treated with 8-10 mm Controlled Expansion diameter VIATORR® (VCX) versus 10 mm diameter first-generation VIATORR® (Legacy) stent-grafts.

**Materials and methods:**

Single-centre retrospective study (January 2015 to March 2024), including 132 adult patients with cirrhosis treated with TIPS due to complications of portal hypertension. Outcomes included post-TIPS new onset overt HE, ascites response, re-bleeding, mortality and portal pressure gradient (PPG) before and after TIPS. Comparisons used Chi square and Fisher´s exact test for categorical variables and Student´s t test or Mann–Whitney test for quantitative variables.

**Results:**

Indication for TIPS was refractory ascites (*n* = 82) and variceal bleeding (*n* = 50). The VCX group (*n* = 85) and the Legacy group (*n* = 47) had similar new onset overt HE: 37% (31/85) vs 43% (20/47), respectively (*p* = 0.31); mortality rates (34% [29/85]) vs 39% [18/47], respectively, *p* = 0.57) and re-bleeding (17% [6/35] vs 20% [3/15], respectively, *p* = 1.00). Median PPG reduction after TIPS was 10 mmHg (7 – 13) in the VCX group and 12 mmHg (9 – 15) in the Legacy group (*p* = 0.02). Subgroup analysis revealed post TIPS overt HE rate of 38% (19/50) in the VCX group vs 53% (17/32) in the Legacy group (*p* = 0.13), with refractory ascites as an indication. Shunt dysfunction rate was 7% (6/85) in the VCX group (stent thrombosis *n* = 6, stenosis or malpositioning *n* = 0) and 0% (0/47) in the Legacy group (*p* = 0.09).

**Conclusion:**

VCX stent grafts induce an immediate lower PPG reduction, which might lead to more stent dysfunctions, but also to a reduction in post-TIPS HE.

## Introduction

Despite technical advances that led to lower transjugular intrahepatic portosystemic shunt (TIPS) complications, hepatic encephalopathy (HE) remains the most frequent adverse event [1–5], without an observable trend towards a decrease in the past years [4–9]. The use of smaller nominal diameter stent-grafts (8 mm instead of 10 mm) has been shown to reduce HE [10–14]. Even if underdilated to 8 mm, 10 mm nominal diameter expanded stent-grafts tend to passively expand to full nominal diameter [15–17]. TIPS with 8 mm diameter stent-grafts frequently lead to higher portal pressure gradient (PPG) (> 12 mmHg) with poor ascites/variceal bleeding (VB) control; whereas TIPS with 10 mm diameter stent-grafts frequently lead to lower PPG (< 8 mmHg) with better ascites/VB control. However, 10 mm diameter stent-grafts have been shown to increase the incidence of post-TIPS HE [18, 19]. The VIATORR® Controlled Expansion (VCX) stent (W.L. Gore & Associates, Phoenix, AZ, USA) was introduced in 2016 to tackle this stent-graft diameter compromise. It has a 8-10 mm nominal diameter that can be underdilated to 8 or 9 mm, due to the presence of an extra sleeve that limits its passive expansion to the full 10 mm diameter [20, 21]. Previous studies have shown contradicting results, regarding HE rates and clinical outcomes post-TIPS with VCX versus Legacy stent-grafts. Some studies showed a reduction of post-TIPS HE with the VCX stent-grafts, whereas others failed to prove so [22–24]. Thus, we retrospectively compared the frequency of post-TIPS HE between patients treated with the VCX stent-graft and the Legacy stent-graft.

## Materials and methods

### Patients and intervention description

A single-center retrospective study including consecutive adult patients (≥ 18 years old) with liver cirrhosis who received TIPS due to complications of portal hypertension at the Interventional Radiology Unit of the Curry Cabral Hospital (Lisbon, Portugal) between January 2015 and March 2024 was conducted. Patients with complicated portal hypertension referred for TIPS placement due to refractory ascites and VB were included. Patients undergoing liver transplantation were censored during follow-up at the time of liver transplant. Exclusion criteria were absent follow-up data, technical failure (inability to place a TIPS stent-graft), portal vein recanalization and TIPS performed in non-cirrhotic patients with portal hypertension. Institutional Review Board approval was obtained for this study. All patients signed an individual informed consent (except for emergent VB) before TIPS.

**Fig. 1:**
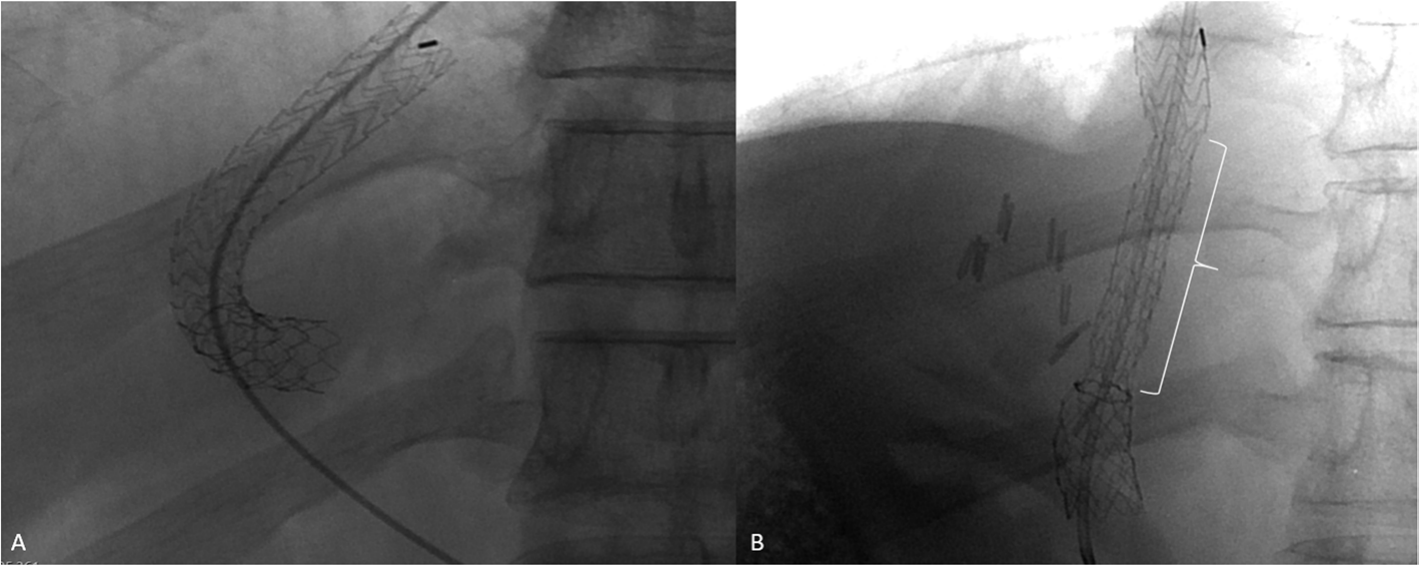
Radiographic images after deployment of the Legacy stent-graft (**A**) and the VCX stent-graft (**B**). Notice the diameter is the same (10 mm) along the Legacy stent-graft but constrained (8 mm) in the VCX stent-graft (highlighted), where an extra-sleeve prevents further passive expansion to the nominal (10 mm) diameter

Two groups were compared: the VCX group (patients who received the 8 mm-10 mm VIATORR® VCX stent-graft) and the Legacy group (patients who received the 10 mm diameter first-generation VIATORR® stent-graft). Patients included in the Legacy group have been previously published [25]. The criteria for patient allocation was time: Legacy group encompassed all TIPS procedures performed between January 2015—May 2018, whereas VCX group included all TIPS procedures performed between May 2018—March 2024. All TIPS procedures were performed by 3 interventional radiologists with 25, 14 and 7 years of expertise as previously described (Fig. 1) [25]. Briefly, a 10-French right jugular access was obtained in all patients and the Rösch-Uchida Transjugular Liver Access Set—RUPS (Cook, Bloomington, IN, USA) was used. After catheterization of the right or middle hepatic vein, portal vein puncture was performed with abdominal ultrasound guidance. All procedures were done under general anesthesia and PPG was measured before and after completion of the intervention. Mechanical ventilation was only used in a minority of patients (highly decompensated patients, in salvage TIPS). PPG measurements were performed using a 4-French Pigtail catheter in the inferior vena cava near the right atrium and in the main portal vein trunk before and after TIPS placement using the same transducer at mid-axillary level.

Initial tract dilatation as well as TIPS dilatation were performed with 8 mm angioplasty balloons for the Legacy group and 6 mm angioplasty balloons for the VCX group. All stents were placed with a cranial end < 1 cm from the inferior vena cava. At this point, after initial stent graft dilatation, another PPG measurement was made and hemodynamic success was defined as a reduction in PPG to < = 12 mmHg or at least 50% reduction of the pre-TIPS gradient. When hemodynamic success was not achieved, the TIPS was dilated to 10 mm in the Legacy group and 7 or 8 mm in the VCX group, progressively, until hemodynamic success.Technical success was defined as the creation of a shunt between the hepatic and portal venous system.

### Primary and secondary endpoints

The primary outcome measured was post-TIPS new onset overt HE (grade II or more in the West Haven scale) [26]. The secondary outcome measures included mortality, ascites response (defined as no further need for paracentesis, despite the presence of clinically detectable ascites), re-bleeding (defined as any event of variceal bleeding after TIPS during follow-up), model for end-stage liver disease (MELD) variation (difference between pre- and post-TIPS MELD score), Child–Pugh score post-TIPS, post-TIPS liver failure (PTLF) (defined as ≥ threefold bilirubin and/or ≥ twofold INR elevation from the baseline within 30 days following TIPS procedure [27]), shunt dysfunction (stent thrombosis, stenosis or malpositioning leading to reduced flow and/or recurrence of portal hypertension complications), technical details (PPG before/after TIPS and PPG variation, expressed as the difference between pre- and post-TIPS measurements; number of attempts of portal vein puncture).

Adverse events (according to CIRSE classification of complications [28]) were compared between the two groups.

### Data analysis

Continuous variables were presented as means ± standard deviations (SD) or medians with interquartile ranges (IQRs), depending on the data distribution. Categorial variables as numbers and percentage of patients. Shapiro–Wilk and Kolmogorov–Smirnov tests were used to assess for normality of the data distributions. Independent samples t-test or Mann–Whitney U test were used to compare normal distributions and skewed distributions of continuous variables, respectively. Chi-square and Fisher´s exact test were used to compare categorical variables, the latter for smaller samples. Chi-squared test was used to compare post TIPS overt HE, the primary endpoint, between both groups. It was also used to compare ascites response, re-bleeding, mortality, PTLF, shunt dysfunction, hemodynamic success and adverse event rates. MELD variation after TIPS and PPG variation were compared between groups using Mann–Whitney non-parametric U test. Statistical significance was set at *p* < 0.05.

Data were analysed using the Statistical Package for the Social Sciences for Windows, version 29.0 (IBM Corp. Released 2023. IBM SPSS Statistics for Windows, Version 29.0.2.0 Armonk, NY: IBM Corp).

## Results

**Fig. 2:**
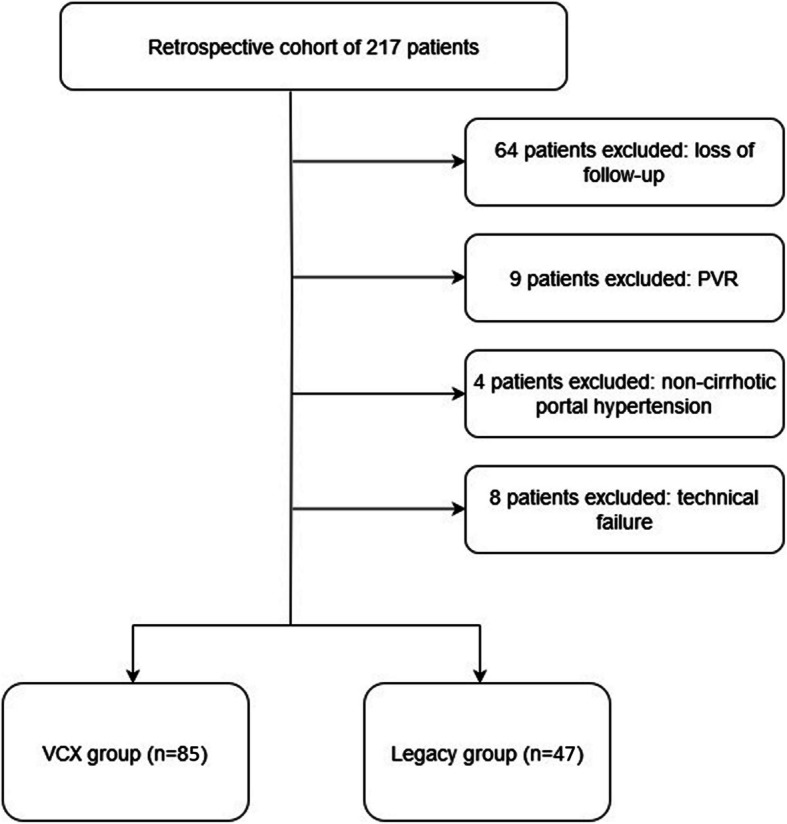
Flow diagram of included patients. PVR—Portal Vein Recanalization; VCX—VIATORR® Controlled Expansion stent-graft. Technical failure (inability to perform TIPS) was reported in 3/47 (6.4%) patients in the Legacy Group and 5/85 (5.9%) patients in the VCX Group (*p* = 0.32)

Flow diagram of included patients is presented in Fig. 2 and baseline data in Table 1. This study included 47 patients in the Legacy Group with a mean follow-up time of 40 months and 85 patients in the VCX group with a mean follow-up time of 31 months (*p* = 0.92). Eighty-two patients had refractory ascites as an indication and 50 patients VB as indication.

**Table 1:** Baseline data comparison between both groups

	VCX stent-graft (*n* = 85)	Legacy stent-graft (*n* = 47)	*p*-value
Age (y)	57.4 ± 9.7	56.4 ± 11.9	0.61
Male gender (n)	64 (75%)	34 (72%)	0.84
Indication for TIPS (n)			0.35
Refractory ascites	50 (59%)	32 (68%)	
Variceal bleeding	35 (41%)	15 (32%)	
Etiology of cirrhosis (n)			0.16
Alcohol	55 (65%)	27 (57%)	
Alcohol + HCV/HBV	13 (15%)	8 (17%)	
Cryptogenic	0 (0%)	4 (9%)	
HCV	7 (8%)	3 (6%)	
PBC	3 (4%)	2 (4%)	
NASH	5 (6%)	2 (4%)	
Wilson Disease	0 (0%)	1 (2%)	
HBV	2 (2%)	0 (0%)	
MELD score	13 (10–17)	13 (10–16)	0.92
Child Pugh score	8 (7–9)	8 (7–9)	0.90
Hemoglobin (g/dL)	8.9 (7.8–11.2)	10 (8.6–12.1)	0.04
Platelets (× 10^9/L)	116 (65 -173)	115 (67–175)	0.96
Total bilirubin (mg/dL)	1.10 (0.69–1.75)	1.29 (0.85–1.86)	0.15
Albumin (g/L)	31 (28–36)	32 (29–38)	0.54
Creatinine (mg/dL)	1.03 (0.73–1.44)	1.11(0.80–1.82)	0.07
Urea (mg/dL)	46 (34–78)	51 (40–74)	0.34
Serum Na + (mEq/L)	137 (134–140)	137 (133–139)	0.34
INR	1.35 (1.15–1.56)	1.28 (1.16–1.40)	0.17
Baseline HE (n)			0.15
No HE	76 (89%)	41 (87%)	
Grade I	4 (5%)	5 (11%)	
Grade II	4 (5%)	0 (0%)	
Grade III	1 (1%)	0 (0%)	
Grade IV	0 (0%)	1 (2%)	
Baseline Ascites (n)	78 (93%)	39 (83%)	0.14

*HBV/HCV* Hepatitis B/C virus, *HE* Hepatic encephalopathy, *INR* International normalized ratio, *MELD* Model for end-stage liver disease, *NASH* Non-alcoholic steatohepatitis, *PBC* Primary biliary cirrhosis, *TIPS* Transjugular intrahepatic portosystemic shunt, *VCX* VIATORR® Controlled Expansion stent-graft

The rate of post-TIPS new onset overt HE was 37% (31/85) in the VCX group and 43% (20/47) in the Legacy group (*p* = 0.31) (Table 2). Mortality rate was 34% (29/85) in the VCX group and 39% (18/47) in the Legacy group (*p* = 0.57). Ascites response rate was 89% (73/82) in the VCX group and 79% (33/42) in the Legacy group (*p* = 0.10). Re-bleeding rate was 17% (6/35) in the VCX group and 20% (3/15) in the Legacy group (*p* = 1.00). Median and IQR MELD variations post-TIPS were 0 (-3–4) in the VCX group and 2 (-1 – 5) in the Legacy group (*p* = 0.08). PTLF rate was 13% (11/85) in the VCX group and 19% (9/47) in the Legacy group (*p* = 0.10). Shunt dysfunction rate was 7% (6/85) in the VCX group (stent thrombosis *n* = 6, stenosis or malpositioning *n* = 0) and 0% (0/47) in the Legacy group (*p* = 0.09).

**Table 2:** Outcome measures between groups

	VCX stent-graft (*n* = 85)	Legacy stent-graft (*n* = 47)	*p*-value
Post-TIPS new onset overt HE	31 (37%)	20 (43%)	0.31
HE after TIPS			0.42
No HE	50 (59%)	26 (55%)	
grade I	16 (19%)	7 (15%)	
grade II	12 (14%)	12 (26%)	
grade III	5 (6%)	2 (4%)	
grade IV	2 (2%)	0 (0%)	
Time until development of HE after TIPS (days)	30 (15–60)	30 (8–120)	0.77
Mortality (n)	29 (34%)	18 (39%)	0.57
Ascites response (n)	73 (89%)	33 (79%)	0.10
Re-bleeding (n)	6 (17%)	3 (20%)	1
MELD variation after TIPS	0 (-3—4)	2 (-1—5)	0.08
Child Pugh after TIPS	8 (7 – 9)	8 (7 – 10)	0.62
PTLF	11 (13%)	9 (27%)	0.10
Shunt dysfunction (n)			0.09
thrombosis	6 (7%)	0 (0%)	
stenosis	0 (0%)	0 (0%)	

*HE* Hepatic encephalopathy, *MELD* Model for end-stage liver disease, *PTLF* Post-TIPS liver failure, *TIPS* Transjugular intrahepatic portosystemic shunt, *VCX* VIATORR® Controlled Expansion stent-graft

PPG gradients before/after TIPS and variation compared between the VCX and Legacy groups can be found in Table 3. Median PPG variation after TIPS was 10 mmHg (7 – 13) in the VCX group and 12 mmHg (9 – 15) in the Legacy group (*p* = 0.02). Hemodynamic success rate was 100% (85/85) in the VCX group and 98% (46/47) in the Legacy group (*p* = 0.36).

**Table 3:** Technical details between both groups

	VCX stent-graft (*n* = 85)	Legacy stent-graft (*n* = 47)	*p*-value
IVC pressure before TIPS (mmHg)	10 (7–12)	10 (7–13)	0.54
Portal vein pressure before TIPS (mmHg)	27 (23–31)	28 (24–32)	0.16
PPG before TIPS (mmHg)	17 (14 – 20)	17 (15 – 22)	0.17
IVC pressure after TIPS (mmHg)	14 (10 – 18)	14 (13–17)	0.13
Portal vein pressure after TIPS (mmHg)	21 (17 – 24)	20 (18 – 24)	0.35
PPG after TIPS (mmHg)	7 (5—9)	6 (4 – 7)	0.05
PPG variation after TIPS (mmHg)	10 (7 – 13)	12 (9 – 15)	0.02
Portal vein puncture attempts (n)	1 (1–2)	1 (1–1)	0.10
Hemodynamic success (n)	85 (100%)	46 (98%)	0.36

*IVC* Inferior Vena Cava, *PPG* Portal pressure gradient, *TIPS* Transjugular intrahepatic portosystemic shunt, *VCX* VIATORR® Controlled Expansion stent-graft

Adverse events rate was 5% (4/85) in the VCX group and 13% (6/47) in the Legacy group (*p* = 0.17) (Table 4).

**Table 4:** Adverse events between both groups

CIRSE grade	VCX stent-graft (*n* = 85)	Legacy stent-graft (*n* = 47)	*p*-value
1	2 (2%)	3 (6%)	0.17
2	0 (0%)	0 (0%)	
3	2 (2%)	3 (6%)	
4	0 (0%)	0 (0%)	
5	0 (0%)	0 (0%)	
6	0 (0%)	0 (0%)	

*VCX* VIATORR® Controlled Expansion stent-graft, Adverse events included inadvertent puncture of the biliary tree (*n* = 4), liver capsule (*n* = 3) or hepatic artery (*n* = 1) and glue migration to the pulmonary arteries during embolization of gastric varices (*n* = 2)

Nine TIPS revisions were performed in the VCX group and the median stent diameter in revision, before balloon dilatation or stent deployment inside the first stent, was 7.9 mm at a median follow up until revision of 171 days (see Supplementary Table 1).

Subgroup analysis was conducted based on the indication for TIPS: either refractory ascites (*n* = 82) or VB (*n* = 50) (see Supplementary Tables 2–7).

In the refractory ascites group, the rate of post-TIPS new onset overt HE was 38% (19/50) in the VCX group and 53% (17/32) in the Legacy group (*p* = 0.13); global HE rate after TIPS was 40% (20/50) in the VCX group and 56% (18/32) in the Legacy group (*p* = 0.11); ascites response rate was 90% (45/50) in the VCX group and 78% (25/32) in the Legacy group (*p* = 0.12). PTLF rate was 8% (4/50) in the VCX group and 13% (4/32) in the Legacy group (*p* = 0.14). Shunt dysfunction rate was 10% (5/50) in the VCX and 0% (0/32) in the Legacy group (*p* = 0.09). PPG gradients before/after TIPS and variation compared between the VCX and Legacy groups can be found in Supplementary Table 6. Median PPG variation after TIPS was 9 mmHg (7 – 13) in the VCX group and 12 mmHg (9 – 13) in the Legacy group (*p* = 0.02).

In the VB group, the rate of post-TIPS new onset overt HE was 34% (12/35) in the VCX group and 20% (3/15) in the Legacy group (*p* = 0.25); global HE rate after TIPS was 43% (15/35) in the VCX group and 20% (3/15) in the Legacy group (*p* = 0.11); re-bleeding rate was 17% (6/35) in the VCX group and 20% (3/15) in the Legacy group (*p* = 0.55). PTLF rate was 22% (7/32) in the VCX group and 36% (5/14) in the Legacy group (*p* = 0.26). Shunt dysfunction rate was 3% (1/35) in the VCX and 0% (0/15) in the Legacy group (*p* = 0.70). PPG gradients before/after TIPS and variation compared between the VCX and Legacy groups can be found in Supplementary Table 7. Median PPG variation after TIPS was 12 mmHg (8 – 13) in the VCX group and 12 mmHg (9 – 16) in the Legacy group (*p* = 0.28).

## Discussion

The use of controlled expansion stent-grafts was associated with a non-significant lower incidence of HE after TIPS when the indication was refractory ascites. There was also a trend for lower PTLF with VCX. There was a slight increase in shunt dysfunction with the VCX that might be associated with lower PPG variations after TIPS deployment. It might be the case that the VCX stent grafts induce a slightly lower reduction of the PPGs, requiring more revisions due to dysfunction, but, at the same time, induce less hepatic decompensation and less HE after TIPS. The clinical success (ascites/VB control) and mortality rates were not affected with the VCX stent grafts.

Subgroup analysis also revealed a tendency towards lower post TIPS overt HE rate and lower post TIPS global HE when refractory ascites was the indication, with higher rates of shunt dysfunction. However, when VB was the indication, both post TIPS overt HE and post TIPS global HE were non-significantly higher in the VCX group (possibly due to the smaller subsample size in this subgroup analysis). It is possible that these results hold for larger cohorts, owing to a tendency towards a more aggressive PPG reduction in VB, with more dilatations, which can lead to overshunting, as shown by similar PPG variations between the VCX and the Legacy groups in the VB subgroup analysis.

It has been shown that the VCX stent grafts show an abrupt and disproportionate decrease in radial resistive force and chronic outward force at an external diameter of 8.3 mm, thus passive expansion to its nominal diameter of 10 mm is not to be expected [29]. Even after dilation of the TIPS to 10 mm, the VCX stent-graft diameter expansion is ‘‘controlled’’ up to nearly 9.3 mm [29, 30]. Even though the VCX stent graft was underdilated with a 6 mm balloon in our study, expansion until a diameter close to 8 mm is expected to occur, at a rate currently unknown. A VCX stent without subsequent balloon dilatation is expected to passively expand until a diameter of between 8.3 and 8.5 mm in most cases [29]. Dell et al. [29] experimental study shows predictability of the expansion of the VCX stent without or with balloon dilatations of 8, 9 or 10 mm. Praktiknjo et al. [22] measured stent diameters in vivo after balloon dilatations of 8 mm, with median values of 8 mm at a median follow-up by CT of 359 days. A 6 mm balloon dilatation (not described in other centres), instead of 8 mm, in the VCX group, can limit the generalizability of our results, and explain to some degree, the higher PPG values immediately after TIPS in the VCX group. The higher rates of occlusion and slightly lower rates of overt HE after TIPS in the VCX group are probably unrelated, as these two outcomes were measured after a mean follow-up of 31 months, permitting VCX passive expansion to a larger diameter, closer to 8 mm. We performed 9 TIPS revisions in the VCX group and, in 8 patients with available 2D images from fluoroscopy, the median diameter of the stent during revision, before intervention, was 7.9 mm at a median follow up of 171 days, giving an estimate of in vivo stent diameter expansion.

When hemodynamic success was not achieved after TIPS deployment in the VCX group, further dilatation with a 7- or 8-mm balloons was performed, thus following the expected known path of stent expansion—until a maximum diameter of 8.6 mm [29]. This is not very different from the expansion with a 6 mm balloon, possibly closer to 7.9 mm, according to our data. Larger cohorts of TIPS patients underdilated to 6 mm, with serial imaging follow-up, are needed to prove this.

Stent geometry is also a matter of debate [31–33], with the only consistent factor being the distance between the cranial end of the stent and the hepatocaval junction, that should be ≤ 6 mm, according to the latest CIRSE standards of practice [34, 35]. Distances longer than 6 mm increase the risk of stenosis [33] and, consequently, lead to higher PPG values and theoretically a more difficult control of ascites/VB. In our center, we placed all our stents with a cranial end < 1 cm from the inferior vena cava and not close enough to compromise liver transplant, so we believe stent position did not bias our results. When tested in vitro, in out of the box conditions at 37ºC, according to Dell et al. [29], the stent does not expand uniformly but assumes a dogbone shape (narrower in the middle third of the stent and wider at the extremities). In our 9 revisions we noticed an attenuation of the initial dogbone shape.

VCX stent dilatation during the procedure to titrate PPG or even if clinical success was not achieved during follow-up would not have been possible with a fixed diameter Legacy stent. The design of this stent graft therefore permits an easier adjustment of the trade-off between portal hypertension relief and overshunting [30].

Utilization of general anesthesia during TIPS creation raises the intra-procedural right atrial pressure compared to conscious sedation [36], thus reducing the PPG. In our procedures, done with general anesthesia, PPG measurements may then be underestimated, but the PPG variation may be the same. Mechanical ventilation, by raising intrathoracic pressures and atrial pressure, can also lead to an underestimation of PPG measurements. Therefore, immediate PPG during TIPS procedure is different from values at 24 h and later. As proven in other studies, PPG rises over time, even when TIPS is performed under local anesthesia. Also, PPG fluctuates in an unpredictable manner in patients suffering from decompensations [37, 38].

Our study lacks PPG measurements 24 h or later after TIPS placement, therefore potentially limiting the clinical relevance of our PPG measurements. However, PPG variation may be the same, as well as the differences in PPG variation between the VCX and the Legacy stent. Pieper et al. [15] reported mean PPG variations of 12.1 mmHg with an 8 mm Legacy stent dilatated to 8 mm. Schepis et al. [17] had mean PPG variations of 10.7 mmHg with 6 mm underdilation of an 8 mm Legacy stent and 14.2 mmHg with a dilatation of 8 mm of an 8 mm Legacy stent (under conscious sedation). Li et al. [39] had mean PPG variations of 12.4 mmHg with a dilatation of 8 mm of an 8 mm Legacy stent (with conscious sedation). Miraglia et al. [20] had mean PPG variations of 9.3 mmHg in a VCX stent underdilated to 8 mm (under general anesthesia). Praktiknjo et al. [22] had mean PPG variations of 10 mmHg in both the VCX and the 10 mm-Legacy stent underdilated groups. Lower stent diameters and underdilations with smaller balloons provide lower PPG variations. It is possible that our lower immediate PPG variation is justified by underdilation with an 6 mm balloon instead of an 8 mm balloon.

Rössle el al showed that most patients reached the treatment goal with stent diameters between 6 and 8 mm. Smaller stents, associated with lower PPG variations, allow gradual dilatation if necessary, preventing excessive pressure reduction, that might reduce post-TIPS overt HE and cardiac dysfunction [40].

The retrospective study of Mansour et al. [24] found similar rates of post-TIPS HE requiring hospitalization between the VCX group and the Legacy group. This was probably due to patient selection (possibly with high MELD scores), as 38% of the VCX stents were dilated to 10 mm during revisions, leaving only 20 in the 8 mm arm for comparison, which is a low number to reach any conclusions. TIPS is recommended earlier in the course of the disease to be beneficial; Kloster et al. [23] found lower (but non-significant) rates of post-TIPS HE (21%, 32%, 49% and 58% at 1, 3, 6 and 12 months respectively for the 8 mm VCX group and 40%, 60%, 80% and 80% for the 10 mm VCX group), but their sample size was small (VCX 8 mm, *n* = 28; VCX 10 mm, *n* = 10); Praktiknjo et al. [22] found significantly lower rates of post-TIPS HE and mortality with controlled-expansion stent-grafts (23% vs 51% and 15% vs 45%), probably because of its prospective nature and an extra arm (VCX fully dilated to 10 mm).

Other limitations to our study include the retrospective non-randomized nature of the data and small sample size. Factors influencing the development of HE after TIPS are multifactorial, including variables such as medical therapy with lactulose/rifaximin use, the presence of a splenorenal shunt, and whether it was treated during TIPS placement [30]. These factors were not specifically analysed in the study, yet they could significantly impact the results.

Time-dependent variables, including HE, stent thrombosis and mortality, would have been better studied using time-to-survival analyses, to account for temporal effects on outcomes.

Liver function tests variations after TIPS were not compared between groups. However, we included surrogates such as MELD and Child–Pugh scores.

In conclusion, our study suggests that VCX stent grafts may induce a lower immediate PPG reduction, especially if underdilated with a 6 mm balloon, which might lead to more stent dysfunctions, but also may reduce post-TIPS HE and hepatic decompensation from overshunting, mainly when refractory ascites is the indication. The VCX and Legacy stent grafts have similar technical and clinical outcomes and adverse event rates. Future studies should define what should be the first-line option to use for TIPS, VCX or Legacy stent grafts.

## Data Availability

Manuscript data and material is available upon request.

## Abbreviations

HE: Hepatic encephalopathy
IQR: Interquartile Range
IVC: Inferior Vena Cava
MELD: Model for end-stage liver disease
PPG: Portal pressure gradient
PTLF: Post-TIPS liver failure
TIPS: Transjugular intrahepatic portosystemic shunt
VB: Variceal Bleeding
VCX: VIATORR® Controlled Expansion stent-graft
